# Molecular determinants of ASIC1 modulation by divalent cations

**DOI:** 10.1038/s41598-024-52845-3

**Published:** 2024-01-28

**Authors:** Yi Liu, Jichun Ma, Renee L. DesJarlais, Rebecca Hagan, Jason Rech, Changlu Liu, Robyn Miller, Jeffrey Schoellerman, Jinquan Luo, Michael Letavic, Bruce Grasberger, Michael P. Maher

**Affiliations:** 1 Neuroscience Discovery, Janssen Research & Development, L.L.C., 3210 Merryfield Row, San Diego, CA 92121 USA; 2 Therapeutics Discovery, Janssen Research & Development, L.L.C., Welsh & McKean Roads, P.O. Box 776, Spring House, PA 19477 USA; 3 Therapeutics Discovery, Janssen Research & Development, L.L.C., 3210 Merryfield Row, San Diego, CA 92121 USA

**Keywords:** Ion channels in the nervous system, Ion transport

## Abstract

Acid-sensing ion channels (ASICs) are proton-gated cation channels widely expressed in the nervous system. ASIC gating is modulated by divalent cations as well as small molecules; however, the molecular determinants of gating modulation by divalent cations are not well understood. Previously, we identified two small molecules that bind to ASIC1a at a novel site in the acidic pocket and modulate ASIC1 gating in a manner broadly resembling divalent cations, raising the possibility that these small molecules may help to illuminate the molecular determinants of gating modulation by divalent cations. Here, we examined how these two groups of modulators might interact as well as mutational effects on ASIC1a gating and its modulation by divalent cations. Our results indicate that binding of divalent cations to an acidic pocket site plays a key role in gating modulation of the channel.

## Introduction

Acid-sensing ion channels (ASICs) are proton-gated cation channels widely expressed in nervous systems^1,2^. At least four genes encode various homomeric (ASIC1a, ASIC1b, ASIC2a, ASIC3, and ASIC4) and heteromeric channels^3,4^. The homomeric ASIC1a is a major proton sensor in the brain and is involved in synaptic function^5–8^ and neuronal injury^9–13^. ASIC3, on the other hand, plays a role in pain perception^14,15^.

Gating of ASIC channels is modulated by divalent cations and small molecules alike. Divalent cations have been shown to stabilize the closed state and/or block open channels^16–21^. However, the molecular determinants of their gating modulation are poorly understood despite the fact that several divalent cation binding sites have been identified^22^. Recently, we reported that two small molecules, JNJ-799760 and JNJ-67869386, bind to a novel site in the acidic pocket and, as with divalent cations, also stabilize the closed state of ASIC1 (chicken ASIC1 and rat ASIC1a)^23^. Here, leveraging the known molecular mechanism of JNJ-799760, we examined the interactions between JNJ-799760 and divalent cations. In addition, we further studied mutational effects on ASIC1a gating and its modulation by divalent cations. Our results indicate that binding of divalent cations to an acidic pocket site plays a key role in gating modulation of ASIC1.

## Results

### Divalent cations and JNJ compounds modulate ASIC1a channel gating kinetics in a similar manner

**Figure 1:**
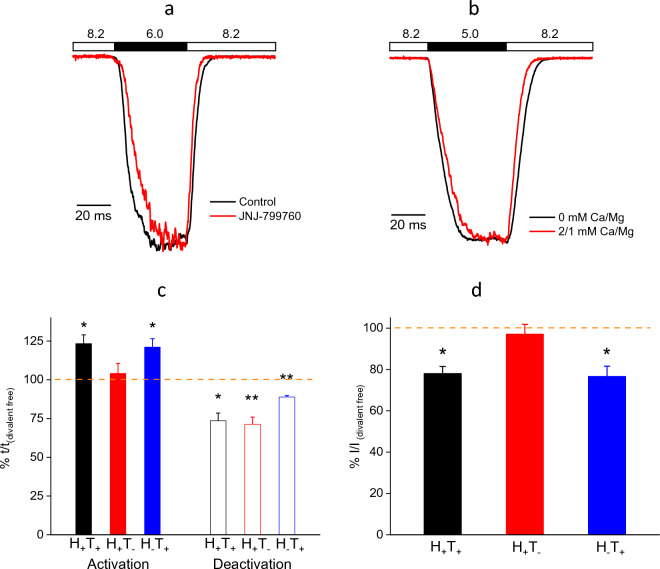
Effects of divalent cations on the activation/deactivation kinetics and peak amplitude of ASIC1a currents. Both JNJ-799760 (100 nM) (**a**) and Ca^2+^/Mg^2+^ (2/1 mM) (**b**) decrease the rate of activation and increase the rate of deactivation. Traces in (**a**) and (**b**) are normalized for the purpose of rate comparisons. JNJ-799760 or 2/1 mM Ca^2+^/Mg^2+^ was present in both holding and test pH buffers. (**c**) Activation (20–80% rise time; solid bars) and deactivation (80–20% decay time; open bars) durations for the indicated divalent cation conditions, normalized to that in divalent-free (both holding and test) pH buffers. H_+_T_+_: 2/1 mM Ca^2+^/Mg^2+^ present in both holding and test pH buffers (n = 3; p < 0.05 for both activation and deactivation); H_+_T_–_: 2/1 mM Ca^2+^/Mg^2+^ in the holding pH buffer and divalent-free in the test pH buffer (n = 5; p > 0.05 for activation and p < 0.01 for deactivation); H_–_T_+_: 0/0 mM Ca^2+^/Mg^2+^ in the holding pH buffer and 2/1 mM Ca^2+^/Mg^2+^ in the test pH buffer (n = 4; p < 0.05 for activation and p < 0.01 for deactivation). The dashed line indicates 100%. (**d**) pH5.0-induced peak current amplitudes for the indicated divalent cation conditions, normalized to that in divalent-free (both holding and test, i.e. H_–_T_–_) pH buffers. For H_+_T_+_, n = 7 and p < 0.05; for H_+_T_–_, n = 4 and p > 0.05; for H_-_T_+_, n = 5 and p < 0.05. The dashed line indicates 100%. All statistical analyses in Fig. 1 are performed using ANOVA.

Previously^23^, we showed that two small-molecules, JNJ-799760 and JNJ-67869386, bind to the same site in the acidic pocket, decrease the rate of activation and accelerate the rate of deactivation in response to step pH changes (also see Fig. 1a for JNJ-799760). Divalent cations (2 mM Ca^2+^ plus 1 mM Mg^2+^ in Fig. 1b) exert similar effects on the channel kinetics. The effects of divalent cations on ASIC1a are rapid and at quasi equilibrium as channels open and close (Fig. 1c and Supplementary Fig. 1). As such, the current amplitude is independent of the concentration of divalent cations in the holding pH buffer and is instead dependent only on their concentrations in the test pH buffer (Fig. 1d). In addition, divalent cations have also been shown to cause fast open channel block of ASIC1a^19^. At 2/1 mM Ca^2+^/Mg^2+^ and test pH of 5.0, this block is ~ 20% in our experiments (Fig. 1d).

**Figure 2:**
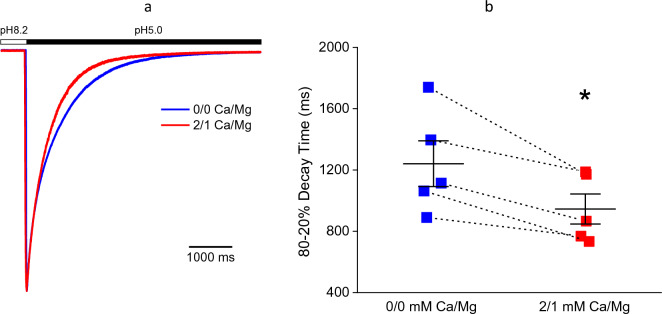
Effects of divalent cations on the kinetics of ASIC1a desensitization. (**a**) Raw traces of pH5.0-induced current (applied for 14 s but truncated for clearer view). Holding pH = 8.2. (**b**) Mean 80–20% current decay time = 1241.0 ± 149.0 ms (0/0 mM Ca^2+^/Mg^2+^; n = 5) and 945.2 ± 98.1 ms (2/1 mM Ca^2+^/Mg^2+^; n = 5; p < 0.05, Student’s t-test). Solid squares represent values from each individual cell.

As with the activation kinetics, the desensitization kinetics are also similarly affected by divalent cations and the JNJ compounds. As shown in Fig. 2a and Fig. 2b, divalent cations increase the rate of open-channel desensitization, as do JNJ-799760 and JNJ-67869386^23^. Furthermore, both groups of modulators decrease the rate of closed-state desensitization^17,23^.

### Divalent cations and the JNJ compounds both stabilize the closed state

**Figure 3:**
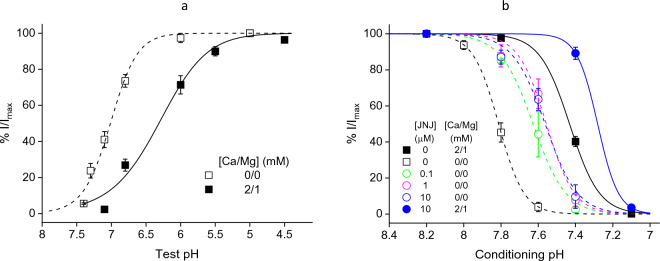
Divalent cations and JNJ-799760 shift the pH dependence of ASIC1a gating in the acidic direction and decrease the effect of one another. (**a**) pH dependence of activation. pH_50_ = 7.01 ± 0.03 (divalent free; open squares; n = 8) and 6.31 ± 0.07 (2/1 mM Ca^2+^/Mg^2+^; solid squares; n = 11). (**b**) pH dependence of steady-state desensitization. In control buffers, pH_50_ = 7.81 ± 0.00 (Ca^2+^/Mg^2+^-free; open squares; n = 4) and 7.43 ± 0.01 (Ca^2+^/Mg^2+^-containing; solid squares; n = 14), respectively. In JNJ-799760-containing buffers, pH_50_ = 7.63 ± 0.01 (0.1 µM, Ca^2+^/Mg^2+^-free; open green circles; n = 3), 7.56 ± 0.02 (1 µM, Ca^2+^/Mg^2+^-free; open magenta circles; n = 3), 7.56 ± 0.02 (10 µM, Ca^2+^/Mg^2+^-free; open blue circles; n = 3), and 7.28 ± 0.00 (10 µM, 2/1 mM Ca^2+^/Mg^2+^; solid blue circles; n = 3), respectively. The Ca^2+^/Mg^2+^-containing control data (solid squares) are from Liu et al.^23^. The holding and test pHs were 8.2 and 5.0, respectively. The conditioning buffer either was nominally free of divalent cations or contained 2 mM Ca^2+^ and 1 mM Mg^2+^. The pH5.0 buffer contained 2 mM Ca^2+^ and 1 mM Mg^2+^ for all experiments. Responses are normalized to those at the holding pH (= 8.2).

Consistent with their effects on channel kinetics, divalent cations cause an acidic shift in the pH dependence of channel activation. As shown in Fig. 3a, the pH_50_ is 7.01 in nominally divalent cation-free buffer vs 6.31 in the presence of 2 mM Ca^2+^ and 1 mM Mg^2+^.

To avoid complications on open channel properties, we took advantage of the fast kinetics of divalent cations on activation (Fig. 1c and d) and studied the effect of divalent cations on steady-state desensitization by holding their concentrations constant in the test pH buffer (containing 2/1 mM Ca^2+^/Mg^2+^). As shown in Fig. 3b, divalent cations and JNJ-799760 both cause acidic shifts in the pH dependence of steady-state desensitization, lowering the pH_50_ from 7.82 in the absence of divalent cations and JNJ-799760 to 7.43 in the presence of 2/1 mM Ca^2+^/Mg^2+^ and 7.66 in the presence of 100 nM JNJ-799760, respectively. This effect was also mirrored by JNJ-67869386 (which shares the same binding site as JNJ-799760), as previously reported^23^.

Taken together, divalent cations, as with JNJ-799760 and JNJ-67869386, destabilize the open and desensitized states in favor of the closed state.

### JNJ-799760 and divalent cations decrease one another’s effects on channel gating

The qualitatively similar effects of divalent cations and the JNJ compounds on gating led us to investigate whether they might be functionally antagonistic to each other. To this end, we expanded the study to also include higher concentrations of JNJ-799760. As shown in Fig. 3b, JNJ-799760 concentration-dependently shifts the pH_50_ for steady-state desensitization to lower pH values in the absence of divalent cations (from 7.81 in the absence of JNJ-799760 to 7.56 in the presence of 10 µM JNJ-799760). This effect is saturated at 1 µM JNJ-799760 as 10 µM JNJ-799760 results in no additional shift. Addition of 2/1 mM Ca^2+^/Mg^2+^ to the 10 µM JNJ-799760 buffer further shifts the pH_50_ to a more acidic value (7.28) beyond that by either 10 µM JNJ-799760 alone (7.56) or 2/1 mM Ca^2+^/Mg^2+^ alone (7.43). However, the amount of acidic shift (ΔpH_50_) by 2/1 mM Ca^2+^/Mg^2+^ in the presence of 10 µM JNJ-799760 (− 0.28) is substantially smaller than that in the absence of JNJ-799760 (− 0.38). Likewise, ΔpH_50_ by 10 µM JNJ-799760 in the presence of 2/1 mM Ca^2+^/Mg^2+^ (− 0.15) is also less than that in the absence of 2/1 mM Ca^2+^/Mg^2+^ (− 0.25).

### The occupancy of a divalent cation binding site is dependent on JNJ-799760

**Figure 4:**
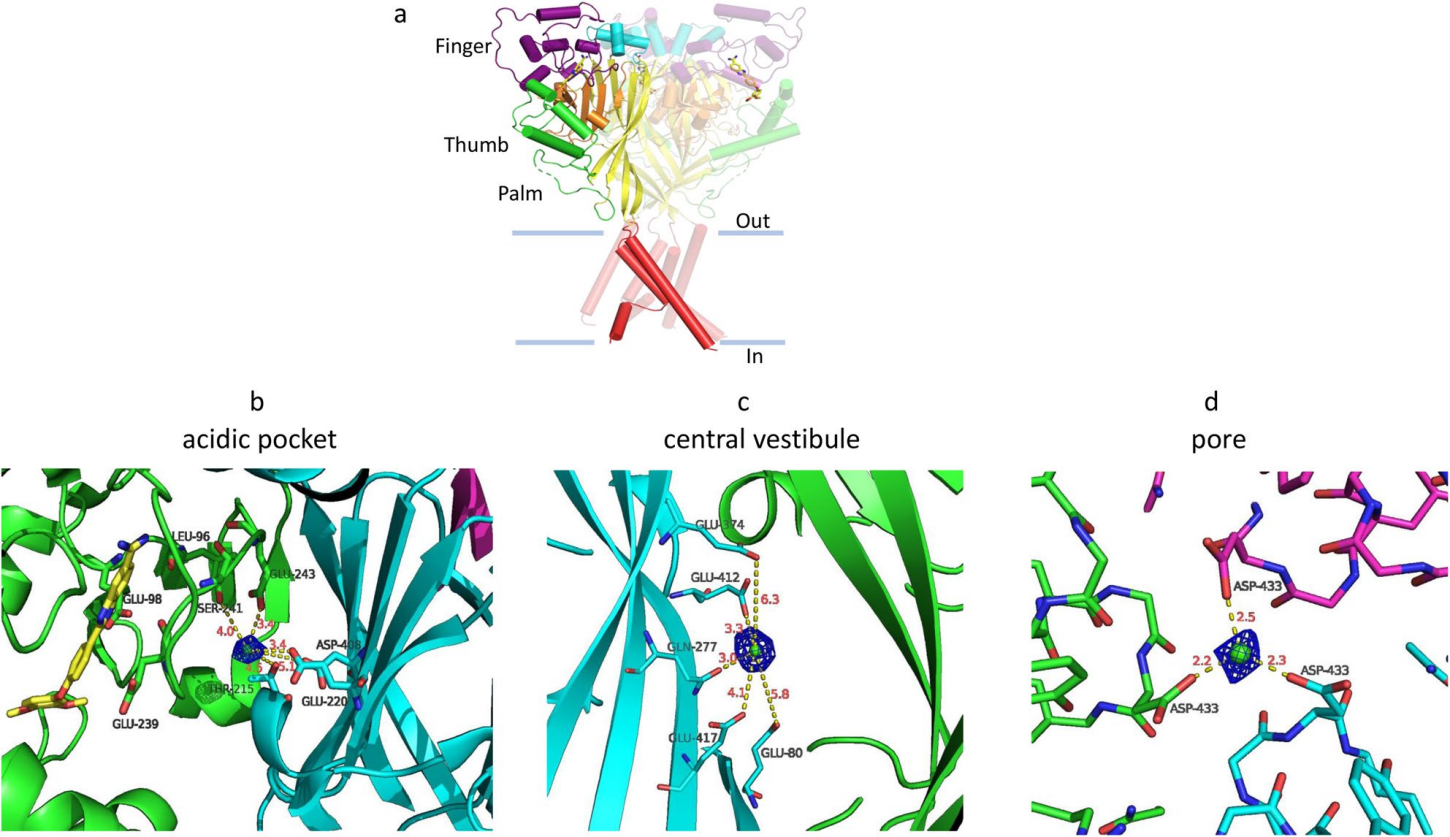
Extra electron densities in ΔASIC1/JNJ-799760. (**a**) Architecture of the ΔASIC1/JNJ-799760 co-crystal structure (viewed parallel to the membrane). One subunit is highlighted with a different color for each domain. JNJ-799760 is in yellow. (**b**) Fo-Fc map contoured at 5.0 σ showing extra electron densities (blue mesh) in the acidic pocket at the interface between subunits A (green) and B (cyan). Numbers (in red) are distances (in Å) between the center of the extra electron density (green sphere) and nearby potential interacting residues (labeled and shown in stick form). JNJ-799760 in subunit A is shown (in yellow). Subunit C is colored in magenta. (**c**) Fo-Fc map contoured at 5.0 σ showing extra electron densities (blue mesh) in the central vestibule near the interface between subunits A (green) and B (cyan). Numbers (in red) are distances (in Å) between the center of the extra electron density (green sphere) and nearby potential interacting residues (labeled and shown in stick form). (**d**) Fo-Fc map contoured at 5.0 σ showing extra electron densities (blue mesh) along the pseudo threefold axis at the channel pore just above the gate (viewed from the extracellular side). Numbers (in red) are distances (in Å) between the center of the extra electron density (green sphere) and D433 (in stick form) from subunits A (green), B (cyan) and C (magenta), respectively.

Recently, we reported a crystal structure of cASIC1 in complex with JNJ-799760 (ΔASIC1/JNJ-799760; PDB code: 6X9H) and showed that JNJ-799760 bound to a novel site in the acidic pocket in the channel’s closed state^23^ (also Fig. 4a and b). Inspection of the ΔASIC1/JNJ-799760 electron density maps in the extracellular domain reveals extra electron densities at two locations (per subunit), one in the acidic pocket (3.4 ~ 5.1 Å from T215, E220, D408 of one subunit and from S241 and E243 of a neighboring subunit; Fig. 4b) and the other in the central vestibule (3.0–6.3 Å from E80, Q277, E374, E412 and E417; Fig. 4c). These locations agree with those from studies of other compound-bound closed-state ΔASIC1 structures from our own group (manuscript in preparation) as well as with those described in the literature^22,24^. In ΔASIC1/JNJ-799760, however, no obvious extra electron densities are present near E98 (Fig. 4b), where a third (i.e. a second acidic-pocket) divalent cation binds in the above-mentioned studies. JNJ-799760 binds near this cation site and indeed, makes a hydrogen bond with the carboxyl side chains of E98 (Fig. 4b), raising the possibility that the close proximity of JNJ-799760 to this site may hinder or preclude cation binding here.

We also observed unallocated electron densities along the pseudo threefold axis in the pore just above the channel gate, ~ 2.5 Å from the oxygens of the D433 side chains (Fig. 4d). Interestingly, no ion binding near D433 has been reported to date in closed-state apo structures.

### Diminished effects of divalent cations on the gating of ASIC1a E97A mutant channels

Previously, binding of divalent cations at the site near E98 was shown to be state dependent in favor of the closed state^22^. As shown above, this cation site is apparently unoccupied in the presence of JNJ-799760 (which interacts with E98 and may preclude cation binding here) even in the closed state. Together with our observation that JNJ-799760 functionally antagonizes gating modulation by divalent cations (Fig. 3b), these results strongly implicate a role of E98 in divalent cation binding and the resulting gating modulation. To further illuminate the contribution of E98, we neutralized the equivalent acidic residue (E97) in rASIC1a from glutamate to alanine and studied the effect of divalent cations on the gating of the mutant channel.

**Figure 5:**
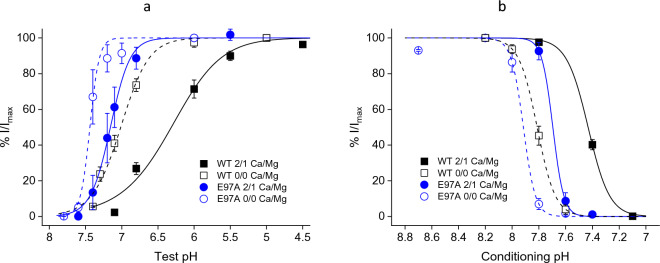
Diminished effects of divalent cations on the gating of rASIC1a mutant, E97A. (**a**) pH dependence of activation in the absence (open symbols) and presence (solid symbols) of 2/1 mM Ca^2+^/Mg^2+^ for wild-type (squares) and E97A mutant (circles) channels. pH_50_ = 6.31 ± 0.07 (WT, Ca^2+^/Mg^2+^ containing; n = 11), 7.01 ± 0.03 (WT, Ca^2+^/Mg^2+^ free; n = 8), 7.16 ± 0.01 (E97A, Ca^2+^/Mg^2+^ containing; n = 6), and 7.44 ± 0.02 (E97A, Ca^2+^/Mg^2+^ free; n = 4), respectively. Responses are normalized to those at pH 5.0. (b) pH dependence of steady-state desensitization in the absence (open symbols) and presence (solid symbols) of 2/1 mM Ca^2+^/Mg^2+^ for wild-type (squares) and E97A mutant (circles) channels. pH_50_ = 7.43 ± 0.01 (WT, Ca^2+^/Mg^2+^ containing; n = 14), 7.81 ± 0.00 (WT, Ca^2+^/Mg^2+^ free; n = 4), 7.70 ± 0.00 (E97A, Ca^2+^/Mg^2+^ containing; n = 3), and 7.92 ± 0.02 (E97A, Ca^2+^/Mg^2+^ free; n = 4), respectively. Responses are normalized to those at pH 8.2, the holding pH. The pH5.0 test buffer contained 2 mM Ca^2+^ and 1 mM Mg^2+^ for all experiments in (**b**). The WT Ca^2+^/Mg^2+^-containing data in (**a**) and (**b**) are from Liu et al.^23^.

The pH dependence of steady-state desensitization of E97A is independent of divalent cations in the test pH buffer (Supplementary Fig. 1), as would be expected from their fast-kinetic effects on channel activation (Fig. 1 and Supplementary Fig. 2) and slower kinetics of desensitization (Fig. 2)^18,22,23^. E97A causes a basic shift of the pH dependence of activation (Fig. 5a) and steady-state desensitization (Fig. 5b) relative to wild-type rASIC1a, consistent with the notion that this residue participates in proton-dependent gating. More importantly, gating modulation by 2/1 mM Ca^2+^/Mg^2+^ is greatly diminished in E97A compared to that in the wild-type channel, supporting the idea that E97 in rASIC1a is involved in binding of divalent cations and stabilization of the closed state—The divalent cation-induced acidic shift, ΔpH_50_, is -0.70 (WT) and -0.28 (E97A) units, respectively, for activation (Fig. 5a) and -0.38 (WT) and -0.22 (E97A) units, respectively, for desensitization (Fig. 5b).

## Discussion

As shown in this and earlier studies, divalent cations and JNJ-799760/JNJ-67869386 exhibit several similarities in their modulation of ASIC1 channels. As with the JNJ compounds^23^, extracellular Ca^2+^/Mg^2+^ ions (1) decrease/increase the rate of ASIC1 activation/deactivation (Fig. 1), (2) slow the rate of closed-channel desensitization^17^, (3) accelerate the rate of open-channel desensitization^19^ (Fig. 2), (4) shift the pH dependence of activation and desensitization to more acidic pH values^17,18^ (Fig. 3), and (5) attenuate tachyphylaxis^25^. One difference is that divalent cations speed recovery from desensitization^17,18^, whereas the JNJ compounds do not alter this rate^23^. The broad similarities raise the possibility of a shared/related molecular mechanism of action for the two groups of modulators.

Studies of cASIC1 apo structures demonstrated that binding to two of the three divalent cation sites in the extracellular domain is state dependent^22^ (favoring the closed over desensitized state), raising the possibility of gating modulation by these cations. One of these sites is positioned near E98 and E239 in the acidic pocket. Intriguingly, we found no evidence of cation binding near E98/E239 in ΔASIC1/JNJ-799760 despite the channel being in the closed state, suggesting that JNJ-799760 may interfere with cation binding at this location. Indeed, the divalent cation (in the apo structure) and JNJ-799760 are both merely ~ 3 Å from E98, with which JNJ-799760 makes a hydrogen bond (The neuropeptide Big dynorphin, which also stabilizes the closed state of ASIC1a, binds at the acidic pocket^26,27^ and interacts with E98 as well, among other residues^27^). Thus, it is possible that JNJ-799760 sterically hinders cation binding to E98. Consistent with this possibility, the divalent cation-induced acidic shift in the pH dependence of desensitization in the presence of JNJ-799760 is significantly decreased (to 0.28 pH units from 0.38 units in the absence of JNJ-799760). This (decreased) value is similar to the divalent cation-induced shift in the mutant channel E97A (0.22 units) in which the divalent cation site at this residue is removed, arguing for a similar role of the mutation and J-799760 (i.e. retarding binding of divalent cations at this locus). Further supporting this idea, neutralization of the equivalent acidic residue in rASIC1a (E97) resulted in large basic shifts of the pH dependence of gating as well as greatly diminished gating modulation by divalent cations (Fig. 5). Qualitatively similar (albeit smaller with less statistical significance) effects on channel activation were also observed for E98Q in cASIC1^22^. Other neutralizing mutations at the equivalent position in cASIC1 and hASIC1a have been shown to similarly cause alkaline shifts^12,22^. Our results, together with the literature reports, strongly indicate that E98 in cASIC1 plays a key role in the binding of, and the resulting gating modulation by, divalent cations.

Additional binding site(s) appear necessary to fully account for the observed gating modulation by divalent cations. This is evident in Fig. 3b, where divalent cations cause an additional shift of pH50 even in the presence of supra-saturating concentrations of JNJ-799760 that prevent cation binding to the site near E98. (Similarly, the residual amount of divalent cation-induced gating shift in E97A, which is comparable to that in the wild-type channel in the presence of JNJ-799760, also argues for the existence of additional site(s)). It has been suggested that binding of divalent cations to the central vestibule site, which is also state dependent, may also be relevant to gating modulation^22^. Indeed, we observe unallocated electron density in the central vestibule site in ΔASIC1/JNJ-799760 consistent with cation binding, being potentially responsible (at least in part) for the surplus effect of divalent cations seen in Fig. 3b.

We also observed unallocated electron densities at the channel gate of ΔASIC1/JNJ-799760 formed by D433. Cation binding at D433 has not been reported in closed-state apo structures, although Cs^+^ ions can bind to this region in open and desensitized states^28,29^. The equivalent residue in rat ASIC1a^19^ and ASIC3^21^ has been shown to participate in open channel block by Ca^2+^. Additional studies will help to illuminate whether this JNJ-799760-induced cation binding contributes to gating modulation.

## Methods

### Whole-cell patch clamp electrophysiology

Chinese Hamster Ovary (CHO) cells stably or transiently expressing rat ASIC1a wild-type or mutant channels were used. The extracellular solutions nominally free of divalent cations contained (in mM): 149 NaCl, 4 KCl, 5 glucose, 10 HEPES. To obtain divalent cation-containing extracellular solutions, 2 mM CaCl_2_ and 1 mM MgCl_2_ were added. The pH was adjusted by titrating either with NaOH or HCl (5 mM MES was added to solutions at pH 6.0 and lower). Pipette electrodes were filled with an intracellular solution containing (in mM): 135 KCl, 4 MgATP, 0.3 Na_2_GTP, 10 EGTA and 20 HEPES, pH 7.2. Currents were digitized at 10 kHz (Digidata 1550B) and lowpass filtered at 2 kHz (Axopatch 200B). Series resistance was 75% compensated. Responses were elicited by rapid perfusion of acidic solutions using the SF-77B Fast-Step Perfusion device (Warner Instruments) for 40 ms once every 30 s (unless indicated otherwise) and recorded till steady state was reached. The holding potential was 0 mV. JNJ-799760 was synthesized in house and solubilized in DMSO as 10 mM stocks.

### Electrophysiology data analysis

Baseline values (i.e. current amplitudes at the conditioning pH) were subtracted to obtain responses evoked by the test pH. Responses were normalized for each cell before averaging. Concentration–response data were fitted to a logistic function of the form: R = (A_1_–A_2_)/(1 + (C/C_0_)^h^) + A_2_, where R is the normalized response, C is either pH or compound concentration, C_0_ is the pH/concentration at which half-maximal response occurs (pH_50_ or IC_50_), h is the Hill coefficient, and A_1_ and A_2_ are constants. The fitted data are shown as solid or dashed curves. Statistical analyses were performed using ANOVA or two-tailed Student’s t-test, as appropriate. Experimental results are reported as mean ± SEM over independent measurements on n different cells. Data fitting and statistical analyses were performed using Origin (Northampton, MA, USA).

### Protein expression/purification, crystallization, and X-ray data collection/processing/ structure determination

These methods were all described in detail in a previous publication^22^.

### Supplementary Information

Supplementary Figures.

## Data Availability

Coordinates of ΔASIC1/JNJ-799760 were deposited to the online database https://www.rcsb.org/ with accession code 6X9H. The source data underlying the graphs and charts presented in the main figures are available from the corresponding author upon reasonable request.
