# Supplementary material for: Molecular determinants of ASIC1 modulation by divalent cations
Source: Sci Rep. 2024 Jan 28;14:2320. doi: 10.1038/s41598-024-52845-3 (PMC10822848; doi:10.1038/s41598-024-52845-3)
Supplement: Supplementary file 1 — Supplementary Figures. [file 41598_2024_52845_MOESM1_ESM.docx]

**Supplementary Information**

**Molecular determinants of ASIC1 modulation by divalent cations**

Yi Liu^1^, Jichun Ma^2^, Renee L. DesJarlais^2^, Rebecca Hagan^1^, Jason Rech^3^, Changlu Liu^1^, Robyn Miller^2^, Jeffrey Schoellerman^1^, Jinquan Luo^2^, Michael Letavic^3^, Bruce Grasberger^2^ & Michael P. Maher^1*^

^1^Neuroscience Discovery, Janssen Research & Development, L.L.C.,

3210 Merryfield Row, San Diego, CA 92121, USA

^2^Therapeutics Discovery, Janssen Research & Development, L.L.C.,

Welsh & McKean Roads, P.O. Box 776, Spring House, PA 19477, USA

^3^Therapeutics Discovery, Janssen Research & Development, L.L.C.,

3210 Merryfield Row, San Diego, CA 92121, USA

*Correspondence to:

Michael P. Maher

Tel: 858-320-3423

Email: [mmaher1@its.jnj.com](mailto:mmaher1@its.jnj.com)

**Supplementary Figure 1. The pH dependence of steady-state desensitization of rASIC1a E97A is independent of divalent cations in the test buffer.** pH_50_ = 7.92±0.02 (H_-_T_-_; n=4), and 7.92±0.00 (H_-_T_+_; n=4), respectively. Test buffer pH=6.0. Responses are normalized to those at the holding pH (=8.2).

**Supplementary Figure 2. The current amplitude of rASIC1a E97A is dependent only on the concentration of divalent cations in the low pH test buffer.** Normalized pH6.0-induced current amplitude for the indicated divalent cation conditions to that in divalent-free holding and test pH buffers (i.e., H_-_T_-_). For H_+_T_+_, n=4 and p>0.05; for H_+_T_-_, n=3 and p>0.05; for H_-_T_+_, n=6 and p<0.05. Holding pH=8.2 or 8.7. All statistical analyses are performed using ANOVA.
